# Indirect modulation of human visual memory

**DOI:** 10.1038/s41598-021-86550-2

**Published:** 2021-03-31

**Authors:** Stas Kozak, Noa Herz, Yair Bar-Haim, Nitzan Censor

**Affiliations:** School of Psychological Sciences and Sagol School of Neuroscience, Tel Aviv University, Tel Aviv, Israel

**Keywords:** Cognitive neuroscience, Learning and memory, Human behaviour

## Abstract

Conditions in which memories become maladaptive have inspired extensive research geared to modulate memory by targeting it directly and explicitly. Given limitations of direct memory modulation, we asked the following: can the target memories be modulated indirectly? To address this question, we uniquely targeted visual memories, and leveraged a paradigm utilizing instructions to either forget or remember newly encoded memories. We used a multi-domain approach, and applied the instructions to embedded verbal information presented during encoding (words), with the intention to indirectly modulate recognition of the target visual context memory itself (pictures). Accordingly, participants were presented with two lists of words, where each word was preceded and followed by pictures. Participants were instructed to either remember or forget the first list of words. As expected, the instruction to either remember or forget the words differentially influenced word memory strength. Importantly, the instruction regarding the words, indirectly modulated picture memory strength. Better memory for words resulted in reduced picture memory strength and vice versa, with the instruction to remember the words reducing picture memory strength. Together with a negative correlation between word and picture memory strength, the results suggest a competition for shared resources between memory for content and context. These findings may open new avenues to indirectly modulate maladaptive memories.

## Introduction

Dysfunctional memories in psychopathology such as posttraumatic stress disorder and depression encouraged research on memory modulation^1^. As part of this endeavor, pharmacological, behavioral, and non-invasive approaches have been utilized to directly influence targeted memories^2,3^. Although considerable progress has been made, reliable modulation of episodic memory in humans still remains elusive^4^. Pharmacological and non-invasive neuromodulation techniques typically lack specificity and are not always suitable for humans^3,5^, whereas behavioral approaches typically result in small effects on memory^6^ and can sometimes enhance memory strength instead of reducing it^7^.

**Figure 1:**
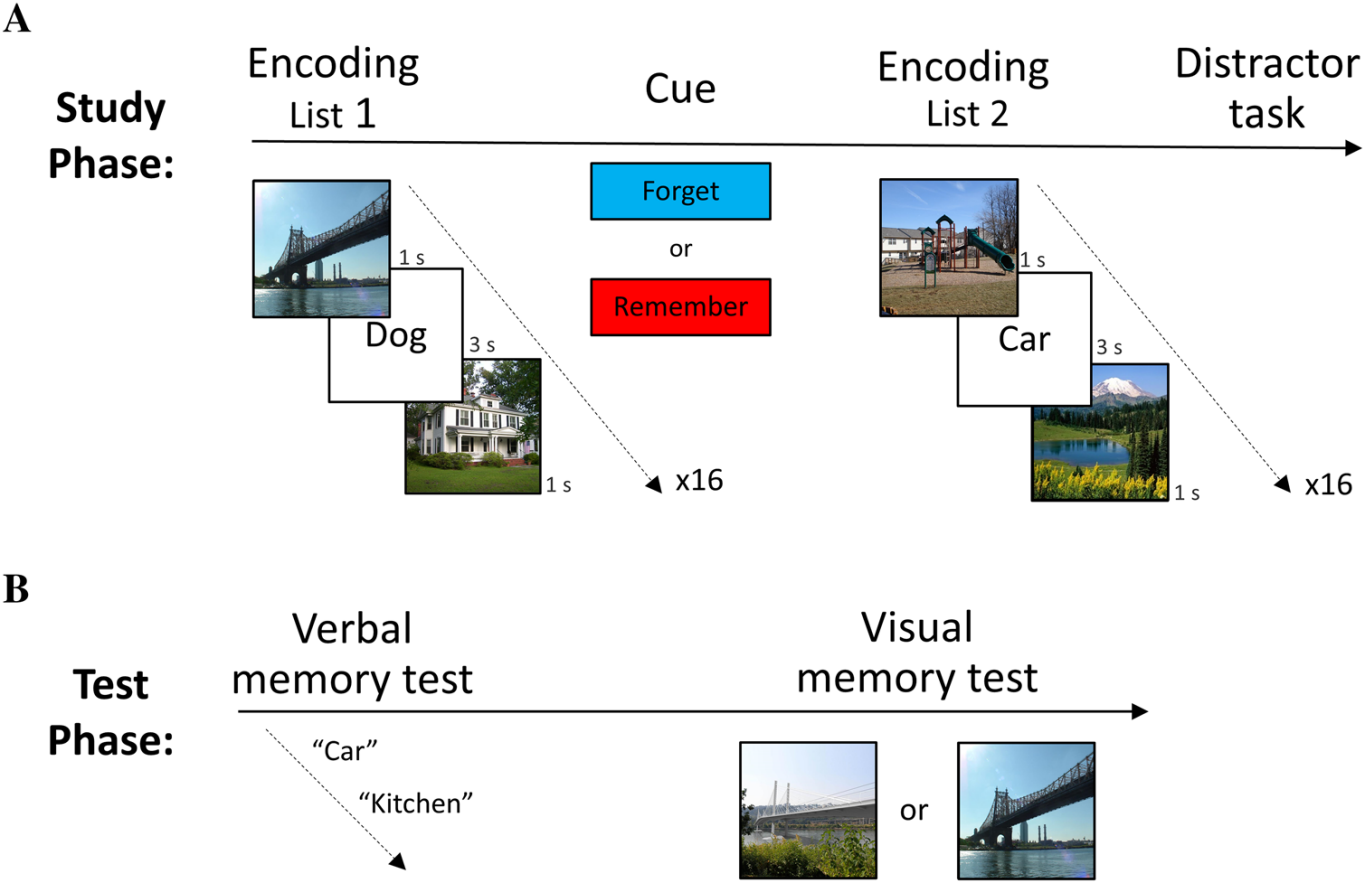
Indirect visual memory modulation experimental design (**A**) Participants were presented with two lists of words intermittently presented between pictures which were the actual target for indirect visual memory modulation. A cue was presented to either forget or remember the first list of words. (**B**) Following encoding, memory strength was tested for the words (direct memory modulation), and the pictures (indirect visual memory modulation). Pictures illustrated are from FIGRIM, a publically available database^54^.

Given the existing challenges associated with direct modulation of memories, we tested whether episodic visual memory could be modulated indirectly. In daily life, content items are typically encoded together with visual contextual scenes, which are then both embedded into the memory trace^8,9^. As such, modulating the encoded item may also inversely influence its contextual frame, potentially due to competition over shared resources^10–12^. Unlike previous studies (e.g.^13^), we applied a multi-domain approach, in which we directly influenced memory in one domain (words), in order to indirectly modulate the target memory in a different domain (pictures). We indirectly targeted visual memories (pictures), as those are highly relevant to real-life memories that can become maladaptive and require modulation. Specifically, we leveraged a directed forgetting (DF) paradigm utilizing instructions to voluntarily forget or remember newly encoded information^14^, commonly geared to modulate the targeted memory, possibly through mechanisms of contextual change^15–20^. However, we applied these direct instructions to embedded verbal information presented during encoding (words), to indirectly modulate recognition of the target visual context memory itself (pictures). Accordingly, participants studied two lists of words embedded within a pictorial context, which was the actual target of memory modulation. Therefore, they received a cue to either forget (n = 20) or remember (n = 20) the words presented in the first list (instructed list 1), without any direct instructions about the contextual pictures in which the list was embedded (Fig. 1) (see “Materials and Methods” section). We reasoned that the cue to forget or remember the words will not only impact memory strength for the words, but will also indirectly modulate picture recognition. Specifically, if remember instructions leading to better memory for words would result in reduced picture memory strength and vice versa, this may support a possibility of competition over shared resources, resulting in indirect picture memory modulation.

## Results

**Figure 2:**
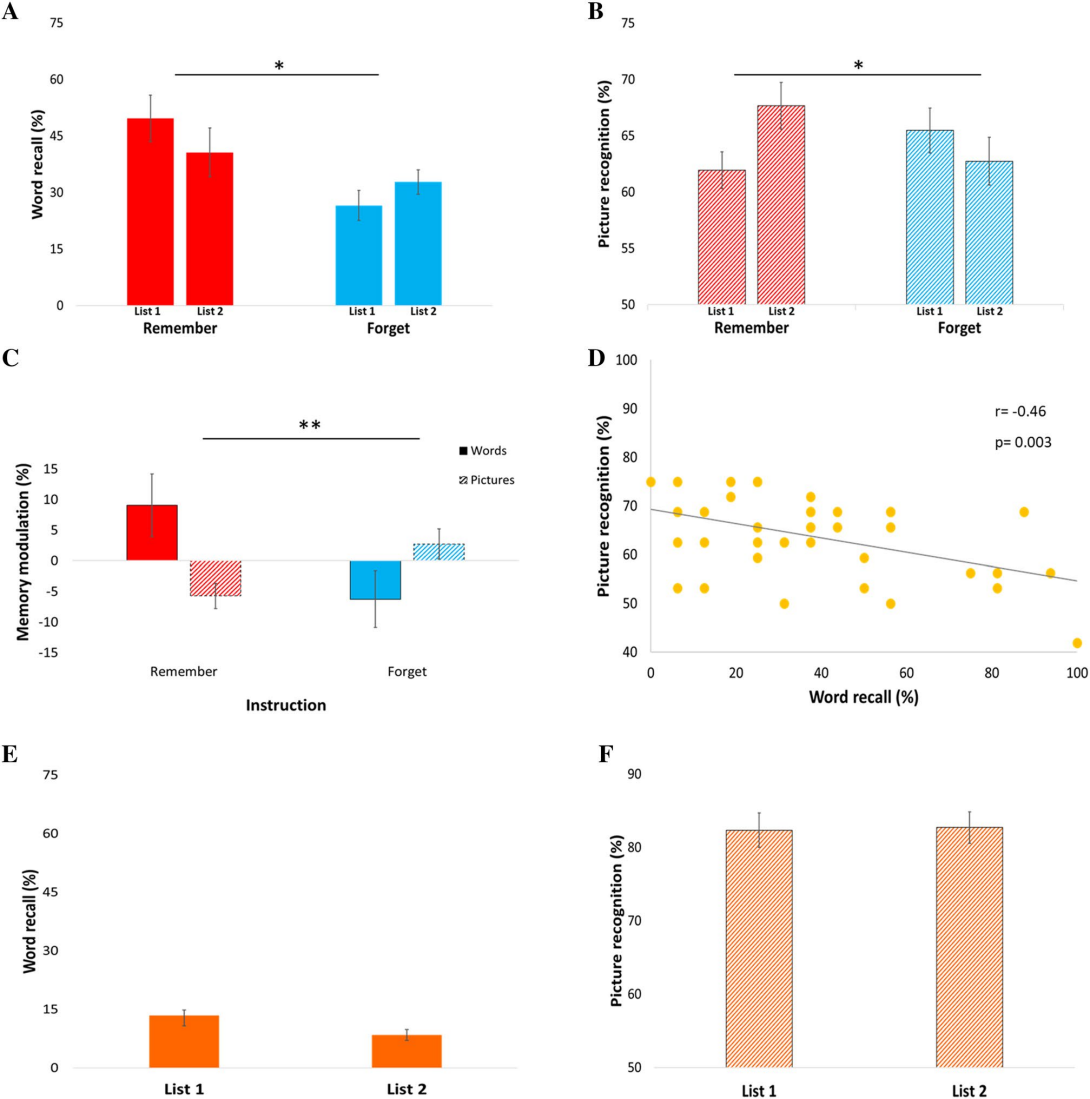
Memory modulation results (**A**) Direct memory modulation. Instructions to either remember (red) or forget (blue) the words differentially influenced word recall, with memory strength greater in the remember than in the forget condition (instructed list 1 relative to list 2). (**B**) Indirect memory modulation. Picture memory strength was lower when instructions were given to remember the words, compared to when instructions were given to forget the words (instructed list 1 relative to list 2). (**C**) Indirect modulation of memory for pictures was in the opposite direction to the memory for the words, with better memory for words under the ‘remember’ instructions resulting in reduced picture recognition and vice versa. (**D**) Consistently, a negative correlation between word and picture memory strength was observed (instructed list 1). (**E**) Control experiment testing for recency effects: Instructions to remember the pictures resulted in a floor effect for word recall, and (**F**) Picture memory strength remained constant across lists. Error bars represent ± 1 Standard error of the mean (S.E.M.) *p < 0.05, **p < 0.005.

Consistent with previous direct memory modulation results, the instruction to either remember or forget the words differentially influenced words recall. Memory strength in the instructed list 1 relative to list 2, was greater in the remember condition than in the forget condition (F(1,38) = 4.906, *p* = 0.033, η^2^_p_ = 0.114, Fig. 2A). A directed forgetting cost effect^14,21^ was confirmed with a post-hoc analysis of instructed list 1 indicating greater memory strength in the remember than in the forget condition (t(38) = − 3.161, *p* = 0.003, d = 1.02).

Can the instructions regarding the words indirectly modulate picture memory strength? Indeed, picture memory strength in the instructed list 1 relative to list 2 was lower when instructions were to remember the words, compared to when instructions were to forget the words (F(1,38) = 7.107, *p* = 0.011, η^2^_p_ = 0.158, Fig. 2B). A post-hoc analysis showed that the instruction to remember the words reduced picture memory strength (instructed list 1 relative to list 2, t(19) = − 2.778, *p* = 0.012, d = − 0.62, Fig. 2B). Of note, these results were not due to the order of the word/picture memory tests (see Supplementary Information).

This indirect modulation of memory for the pictures was thus in the opposite direction to the memory for the words. To directly test the inverse relation between word and picture memory strength, we included in the same model both the words and the pictures, by computing a memory modulation index (instructed list 1 relative to list 2). The results indicate a significant interaction between memory type (word/picture) and instruction (remember/forget), (F(1,38) = 9.180, *p* = 0.004, η^2^_p_ = 0.195, Fig. 2C), such that better memory for words resulted in reduced picture recognition and vice versa. A negative correlation between word and picture memory strength was also evident (instructed list 1, r = − 0.46, *p* = 0.003, Fig. 2D). Together, the results indicate that the instruction to either remember or forget the words indirectly and inversely modulated picture memory strength.

A possible alternative explanation to these results is that lower picture recognition in list 1 relative to list 2 reflects a recency effect^22,23^. Although application of a distractor task as used in our study typically eliminates recency effects^15,18,24^, we nonetheless conducted a control experiment to address this possibility. In the control experiment, participants encoded the same lists, but were instructed to directly remember the pictures instead of the words. As expected, there was a floor effect for word memory strength (Fig. 2E). Importantly, picture memory strength remained constant across lists (instructed list 1 relative to list 2, t(19) = − 0.179, *p* = 0.860, d = − 0.03, Fig. 2F), supporting the notion that memory modulation of picture recognition was driven by the instructions regarding the words, rather than reflecting a recency effect.

## Discussion

The results indicate that visual memories, often relevant to daily life scenarios, can be indirectly modulated. By leveraging a manipulation designed to modify newly encoded memories^14,21^, but applying it in a multi-domain approach to embedded verbal information presented during encoding (words), we were able to indirectly modulate the targeted visual context memory (pictures). While the instruction to forget or to remember the words directly influenced word memory strength, it also indirectly modulated picture memory strength. Better memory for words resulted in reduced picture recognition. Consistently, a negative correlation was observed between word and picture memory strength. An additional control experiment ruled out the possibility that the indirect picture memory modulation resulted from a recency effect. Taken together, the current results support a competition between memory for content and context, suggesting that these two types of memories rely on common neuro-cognitive resources.

The suggestion that the mechanism underlying indirect memory modulation is part of a competition between content and context memories is consistent with previous studies. Emotionally arousing stimuli are often encoded at the cost of encoding the surrounding information^25–27^, suggesting content and context competition over memory encoding resources. Neural evidence supports the interdependency between memory for content and its surrounding context. In an fMRI experiment, instructions to remember the words was found to elevate the neural activity associated with their visual context^20^. Although these results were interpreted as increased representation of the contextual information under the remember instructions^20^, a recent non-monotonic plasticity model proposes that to-be-forgotten memories actually have higher neural activity compared to remembered memories^28^, supporting the possibility of an inverse relation between content and its surrounding context.

Memory for context and content was suggested to be represented in the parahippocampal and perirhinal cortex respectively, two brain regions that are anatomically connected^29^, and may therefore share overlapping resources. In line with this suggestion, a recent transcranial magnetic stimulation study showed that inhibition of lateral occipital cortex activity (LOC; related to object processing) improves scene categorization accuracy via an increase in parahippocampal place area activity (PPA; related to scene processing)^10,11^.

Importantly, our experimental paradigm suggests that memory strength trade-off between content and context can be manipulated through post-encoding processes. The instructions were given for the entire study list after it had already been perceived and encoded without interruption (Fig. 1), suggesting that our manipulation affected post-encoding memory processes^30,31^ and was not driven by immediate modulation of online perception or working memory^32–34^. In addition, our results do not seem to indicate that the instructions to remember or forget had a general effect on attention, since these instructions influenced memory for words and pictures in opposite directions. Namely, the remember instructions did not enhance overall memory, but rather increased only one type of memory while decreasing the other, and vice versa for the forget instructions.

Of note, methods for indirectly influencing encoded memories have previously been utilized, focusing on online rather than post-encoding processing. For example, previous studies applied the Think No-Think paradigm (TNT) using a cue during word presentation signaling participants to avoid thinking about the presented word. By inserting unrelated ‘bystander’ pictures between the suppressed words, it was found that thought suppression also decreased memory for the adjacent bystander items, forming an amnesic shadow affect^35,36^. These results highlight that online compared to post-encoding memory modulation processes may involve different mechanisms. Correspondingly, TNT effects were suggested to rely on right dorsolateral prefrontal cortex (DLPFC) engagement coupled with hippocampus suppression, while the list-method DF was found to involve left DLPFC together with hippocampus uncoupling and alpha/beta frequency phase synchrony reduction^37^.

While previous DF experiments found increased memory for list 2 items in the forget condition (termed a benefit effect), such an effect was absent in the current study. Several reasons can explain these results. First, when participants are required to recall words from list 1 prior to list 2 the benefit effect is often reduced^38^. Although given a free choice subjects may tend to recall list 2 first^39^, participants in our study did not have any consistent tendency to recall items from list 2 before items from list 1 (see Supplementary Information), possibly reducing our ability to detect a benefit effect across subjects. Second, the usage of a relatively long word list, as the one used in this study, could reduce the benefit effect^40^. Third, we cannot exclude the possibility that a different encoding strategy was used when participants learned list 2 relative to list 1, a factor that was also indicated to influence the benefit effect^41^. Finally, the insertion of contextual pictures in our design might have elevated subjects’ cognitive load and reduced the benefit effect^42^.

We designed the visual memory test based on previous studies of forced choice recognition, geared to assess visual memory fidelity^43^ and not only retrieval probability^44,45^. Nevertheless, the divergent methods for measuring memory across words and pictures may constitute a limitation of this study, and it remains to be determined whether the effects observed here are specific to recognition memory or can be extended to freely-recalled memory retrieval, relevant to additional memory domains. In addition, similarity manipulation of the foil pictures can be used to tap into different aspects of visual memory, for example, whether the effects are specific to coarse categorical or fine pictorial representations^44,45^.

Our experimental approach was unique and divergent from previous studies^13,18,30^ in several aspects. First, rather than applying stimuli within a single domain, we chose a multi-domain approach in which we directly influenced memory in one domain, in order to indirectly modulate the target memory in a different domain. Second, we targeted visual memories, since visual memories are highly relevant to real-life memories which can become maladaptive. Third, the participants were not required to judge or direct attention to any aspect of the indirectly modulated memory (the pictures), contrary to explicit encoding processes and a classical DF effect^13,46^. Therefore, our approach reflects a first of its kind indirect and inverse modulation of the targeted visual memory. While DF list-methods designs commonly influence only recall memory^47^, modulation of recognition memory could be evident when associations between the studied items are weak^48^. Consistently, the multi-domain approach utilized here may involve weaker associations between visual and verbal domains, enabling modulation of visual recognition memory.

In summary, by applying indirect memory modulation, current challenges in the field related to direct memory modulation may potentially be overcome. Understanding the mechanisms by which target memories can be indirectly modulated could set the stage for new treatment approaches for clinical disorders such as posttraumatic stress disorder, in which persistent memories of the traumatic event are maladaptive. The current results provide an important stepping stone for research extending the current application to both emotion laden scenes and to clinically relevant populations.

## Materials and methods

### Participants

Sixty-one volunteers (43 females, *M*_*age*_ = 24.98 years*, SD* = 3.96, *Range* = 19–38) were recruited through online and printed advertisements. Exclusion criteria were self-reported neurological, physical, or mental disorders. All participants reported at least 6 h of sleep at the night before the experimental session. One participant was excluded due to difficulty in understanding task instructions. Sample size was determined a priori based on an averaged reported effect size (*η*^2^_p_ = 0.09, power = 0.9) found in previous list-method directed forgetting studies^49,50^. Participants were provided written informed consent prior to testing and were compensated $20 or course credit. The Tel Aviv University Institutional Review Board approved the study, and all methods were performed in accordance with the relevant guidelines and regulations.

### Procedure

Participants performed a modified version of the DF paradigm. Unlike previous DF experiments, the neutral words (16 per list, see Supplementary Information)^51,52^ were embedded within a neutral pictorial context (32 per list)^53–55^, creating a picture-word-picture triplet (Fig. 1). Words were presented for 3 s, and pictures for 1 s. Inter-stimulus-interval (ISI) between triplets was 3 s. To minimize any semantic association biases between words and their surrounding pictures, we randomized both words and pictures across lists.

During debriefing participants were instructed to read the following instructions: "In the following experiment we will ask you to study two lists of words. Each word will appear for a few seconds, and will be preceded and followed by a picture. Your task is to *remember the presented words*, as your memory for these words will be tested at the end of the experiment." These instructions were repeated out loud by the instructor.

After studying the first list, each participant received a cue to either forget or remember the first list of words. Subjects in the remember group received the following written instructions: "The list of words you have just seen was the first part of the study phase. Please *try to remember this list*". The forget group received the following written instructions: "The list of words you have just seen was a practice list. Please *try to forget this list*". Both groups were instructed to remember the second list of words. To reduce recency effects, participants were asked to solve arithmetic problems for 1 min as a distractor before the beginning of the test phase^15,24^.

The first test phase was delivered immediately following the distractor task and comprised of a free recall for words and a picture recognition test (Fig. 1B). In the free recall test, participants were asked to write as many words as they can remember from both studied lists, in any order, including those that they had been instructed to forget. This test was not time limited. In the picture recognition test, participants performed a two-alternative forced choice recognition test in which they saw simultaneously two similar scene images from the same category and had to decide which of the two was shown during the study phase. Subjects were asked to answer as quickly and as accurately as possible. Each test trial ended after the participant's response. The order of free recall and picture recognition tests was counterbalanced across participants.

To assure the pictures were perceived as neutral, participants returned to the lab one day following the study phase and rated the valence of the study pictures^56^. Subjects were exposed to all studied neutral pictures (64 pictures) as well as to new 32 pictures with a negative valence (lower than 3 on a 1–9 Self-Assessment Manikin scale) taken from the IAPS database^56^. A pilot study indicated that neutral ratings are more consistent when negative items are also shown (see Supplementary Information).

### Data analysis

Percent of correct responses was calculated for each list of words and pictures. Data points falling above two standard-deviations from the mean in each experimental condition were winsorized to the highest score inside the range of − 2 < Z < 2^57^. Trials with response time lower than 300 ms (< 1%) were excluded from further analysis.

A two-way mixed ANOVA was calculated for word or picture memory strength, with instruction (remember/forget) as a between-subject factor and list (list 1, list  2) as a within-subject factor. To assess the relation between word and picture memory modulation across lists, a two-way mixed ANOVA was calculated with test type (word/picture) as a within-subject factor and instruction (forget/remember) as a between-subjects factor. A Pearson correlation was calculated between word recall and picture recognition for the instructed list.

## Supplementary Information

Supplementary Information
