# Supplementary material for: Indirect modulation of human visual memory
Source: Sci Rep. 2021 Mar 31;11:7274. doi: 10.1038/s41598-021-86550-2 (PMC8012571; doi:10.1038/s41598-021-86550-2)
Supplement: Supplementary file 1 — Supplementary Information [file 41598_2021_86550_MOESM1_ESM.pdf]

# SUPPLEMENTARY INFORMATION

## Indirect modulation of human visual memory

Stas Kozak*, Noa Herz*, Yair Bar-Haim, Nitzan Censor.

*These authors contributed equally to this work

School of Psychological Sciences and Sagol School of Neuroscience, Tel Aviv University, Tel Aviv, Israel.

Corresponding author: Nitzan Censor

**Email:** censornitzan@tauex.tau.ac.il

To rule out a test order effect, the order of tests (words/pictures) was counterbalanced across participants. A two-way ANOVA on tests order and instruction did not yield a significant main effect ($F(1,36)=0.002$, $p=.962$, $\eta^2p=0.001$) nor an interaction effect for word recall ($F(1,36)=0.517$ $p=.477$ $\eta^2p=0.014$). Similarly, there was no main effect ($F(1,36)=1.581$, $p=.217$, $\eta^2p=0.042$) nor interaction for picture recognition ($F(1,36)=0.057$, $p=.813$, $\eta^2p=0.002$).

To test recall output order in the forget condition, we calculated the percentage of participants beginning their free recall with list 2 words. 55% of the participants started their recall with a word from list 2, while 45% started their recall with a word from list 1 [39].

Individuals' ratings confirmed that the pictures were perceived as neutral (greater than 3 on a 1-9 Self-Assessment Manikin scale, see Materials and Methods) both in the main

experiment (M=5.6, SE=0.06) ($F(1,38)=1013.696$, $p<0.001$, $\eta^2 p=0.964$), and in the control study (M=5.57, SE=0.12) ($t(19)=15.703$, $p<0.001$, $d=3.51$).

The words used in the current study were divided into pairs matched by valence (Mean=2.11, SD=0.84, Range=0.71-3.88 on a 0-30 scale) [51], number of syllables (Mean=2.34, SD=0.48, Range=2-3), number of letters (Mean=4.13, SD=0.71, Range=3-5) and frequency of appearance in the Hebrew language [57]. Each word from these word-pairs was randomly allocated to one of the two study lists (each list consisted of 16 words). To avoid semantic overlap between presented words and pictures, the studied words were of objects with minimum relation to the pictures (scenes).
